# Unlocking the Potential of Wear Time of a Wearable Device to Enhance Postpartum Depression Screening and Detection: Cross-Sectional Study

**DOI:** 10.2196/67585

**Published:** 2025-05-23

**Authors:** Eric Hurwitz, Samantha Meltzer-Brody, Zachary Butzin-Dozier, Rena C Patel, Noémie Elhadad, Melissa A Haendel

**Affiliations:** 1 Department of Genetics University of North Carolina at Chapel Hill Chapel Hill, NC United States; 2 Department of Psychiatry University of North Carolina School of Medicine Chapel Hill, NC United States; 3 Division of Biostatistics School of Public Health University of California, Berkeley Berkeley, CA United States; 4 Department of Infectious Disease University of Alabama at Birmingham Birmingham, AL United States; 5 Department of Biomedical Informatics Columbia University Irving Medical Center New York, NY United States

**Keywords:** wearable device, All of Us, postpartum depression, Fitbit, wear time, screening

## Abstract

**Background:**

Postpartum depression (PPD) is a mood disorder affecting 1 in 7 women after childbirth that is often underscreened and underdetected. If not diagnosed and treated, PPD is associated with long-term developmental challenges in the child and maternal morbidity. Wearable technologies, such as smartwatches and fitness trackers (eg, Fitbit), offer continuous and longitudinal digital phenotyping for mood disorder diagnosis and monitoring, with device wear time being an important yet understudied aspect.

**Objective:**

We aimed to suggest that wear time of a wearable device may provide additional information about perinatal mental health to facilitate screening and early detection of PPD. We proposed that wear time of a wearable device may also be valuable for managing other mental health disorders.

**Methods:**

Using the *All of Us* Research Program dataset, we identified females who experienced childbirth with and without PPD using computational phenotyping. We compared the percentage of days and number of hours per day females with and without PPD wore Fitbit devices during prepregnancy, pregnancy, postpartum, and PPD periods, determined by electronic health records. Comparisons between females with and without PPD were conducted using linear regression models. We also assessed the correlation between Fitbit wear time consistency (measured as the maximum number of consecutive days the Fitbit was worn) during prepregnancy and PPD periods in females with and without PPD using the Pearson correlation. All analyses were run with Bonferroni correction.

**Results:**

Our findings showed a strong trend, although nonsignificant after multiple testing correction, that females in the PPD cohort wore their Fitbits more than those in non-PPD cohort during the postpartum (PPD cohort: mean 69.9%, 95% CI 42.7%-97%; non-PPD cohort: mean 50%, 95% CI 25.5%-74.4%; *P*=.02) and PPD periods (PPD cohort: mean 66.6%, 95% CI 37.9%-95.3%; non-PPD cohort: mean 46.4%, 95% CI 20.5%-72.2%; *P*=.02). We found no difference in the number of hours per day females in the PPD and non-PPD cohorts wore their Fitbit during any period of pregnancy. Finally, there was no relationship between the consistency of Fitbit wear time during prepregnancy and PPD periods (*r*=–0.05, 95% CI –0.46 to 0.38; *P*=.84); however, there was a trend, though nonsignificant, in Fitbit wear time consistency among females without PPD (*r*=0.25, 95% CI –0.02 to 0.49; *P*=.07).

**Conclusions:**

We hypothesize that increased Fitbit wear time among females with PPD may be attributed to hypervigilance, given the common co-occurrence of anxiety symptoms. Future studies should assess the link between PPD, hypervigilance, and wear time patterns. We envision that wear time patterns of a wearable device combined with digital biomarkers such as sleep and physical activity could enhance early PPD detection using machine learning by alerting clinicians to potential concerns and facilitating timely screenings, which may have implications for other mental health disorders.

## Introduction

### Background

The rise of wearable device ownership, such as Fitbits, has led to significant advancements in the realm of digital phenotyping [1]. Because wearables can be used to monitor the same individual in a continuous and longitudinal manner, their use for personalized medicine is exciting, especially for mental health where individualized tools for diagnosis and treatment monitoring are lacking. Digital biomarkers from wearables are collected in a passive manner in nonclinical settings, thus enabling these devices to offer potential enhancements to several clinical aspects of the mental health care continuum [2].

Postpartum depression (PPD) is a mood disorder that is one of the most common complications of childbirth [3]. PPD has significant implications for maternal morbidity, associations with developmental delays for the child, and incurs significant costs to society [4-7]. Because PPD is a highly heterogeneous condition and often stigmatized, many patients go undiagnosed [8]. One significant issue with PPD is that most women do not receive sufficient screening, as only about 31% of women with PPD receive a diagnosis [4]. As noted by Cox et al [4], there are reliable screening instruments for PPD (eg, the Edinburgh-Postnatal Depression Scale [EPDS]) [9] and specific treatments for PPD (eg, brexanalone and zuranolone) [10,11]; yet screening and diagnosis of PPD lag behind and novel approaches for PPD detection are direly needed.

Wearables have provided an opportunistic route for enhanced behavioral phenotyping during pregnancy and in the postpartum period, including for PPD [2,12,13]. Given the underdiagnostic rate of PPD, readily available consumer wearables may aid in its early detection due to their rise in ownership and passive data collection, thereby improving patient outcomes. For example, our recent work demonstrated that individualized machine learning (ML) models using digital biomarkers (heart rate, physical activity, and energy expenditure) from a Fitbit were able to distinguish between 4 phases of pregnancy, including prepregnancy, pregnancy, postpartum, and during PPD [12]. Wearable devices have also been shown to predict whether a woman will experience preterm birth using only 1 week of activity and sleep data [13]. In addition, studies have demonstrated that activity intensity distribution during the day, resting heart rate, and heart rate variability captured from a wearable device were predictive of maternal loneliness, which is associated with PPD [14]. Collectively, these studies highlight a relationship between digital biomarkers and perinatal mental health, suggesting that wearables may enhance longitudinal monitoring.

While it has been shown that digital biomarkers from wearables, such as Fitbit, combined with ML can provide insight into mental health conditions, patterns of wear time remain relatively unexplored. Previous studies exploring wear time of a wearable device have mainly taken place in the human-computer interaction field in a general population and disease-agnostic setting [15-19]. A few studies have looked at wear time behavior in the context of biomedical research, but only in a limited capacity. For instance, analyses from the Framingham Heart Study suggest that higher depressive symptoms are associated with lower smartwatch use, defined as wearing the device for more than 5 hours at least one day of the week. The authors suggest this observation is due to the link between motivation and depressive symptoms, where individuals are less likely to engage with a smartwatch for health-related activities such as tracking daily steps or promoting healthy lifestyle behaviors [20]. While this result posits a relationship between device wear time and mental health, there is a need to explore wear time of a wearable device in pregnancy cohorts, which are heterogeneous and constantly changing, making it difficult to identify potential screening tools and biomarkers.

### Objectives

In this study, we sought to demonstrate the value of wear time of a wearable device as an additional insightful digital biomarker for mental health. Our previous work demonstrated that several digital biomarkers were altered during PPD relative to other periods [12]; therefore, we next wanted to assess differences in wear time of a wearable device across multiple periods of pregnancy. We leveraged the *All of Us* Research Program (AoURP) dataset, a longitudinal, observational dataset with several health-related data types, including electronic health records (EHRs), surveys, physical measurements, and Fitbit data [21]. To highlight the potential value of Fitbit wear time in facilitating early detection of PPD, we characterized differences in wear time between females with and without PPD. We proposed that wear time of a wearable device may contribute to serving as a clinically informative biomarker to help facilitate early detection of mental health disorders in a continuous, passive, and nonclinical setting. For PPD specifically, gaining insight into wearable behavior patterns could offer valuable understanding of perinatal mental health, potentially enhancing screening and diagnosis in real-world settings.

## Methods

### Data Sources and Platforms

Data in this study leveraged the AoURP Controlled Tier version 7 dataset. Analysis was conducted using the AoURP Researcher Workbench cloud platform. All phenotyping and data analysis were conducted using R software (R Foundation for Statistical Computing). Fitbit data in the AoURP operates under a bring-your-own-device model, where participants who consent to participate in the study share data from their device that they already own [22].

### Study Setting, Clinical Setting, and Recruitment Procedures by AoURP

AoURP is a longitudinal, observational study [21]. The data used in this study were obtained from the AoURP, which was responsible for all study and recruitment procedures described in the AoURP Operational Protocol [23]. To briefly summarize study and recruitment procedures from the protocol, anyone who lives in the United States (or territory of the United States) is eligible to enroll in the AoURP. The program emphasizes recruitment of minority populations that have been underrepresented in biomedical research historically. Specific inclusion criteria consist of (1) adults ≥18 years with the ability to provide consent and (2) individuals who currently reside in the United States. The only exclusion criteria include individuals who are incarcerated at the time of enrollment. Recruitment is conducted through targeted advertising (ie, print flyers, brochures, posters, Television, radio, web, mobile, billboards, bus advertisements, email, and snail mail); personal interest groups (ie, social media, community events, and press coverage); and directly at health care provider organizations (HPOs) or direct volunteer (DV) partner sites (ie, waiting areas, regular course of clinical care at HPOs, local informational events, and regional informational events organized by research program awardees, HPOs, or DV partners). Participants enroll to participate in the program through the *All of Us* website or a smartphone app, go through electronic consent modules, and watch explanatory videos with text, icons, and formative questions. Once consented, participants are given baseline health surveys that each take about 15 minutes to complete. In addition, participants then provide authorization to share EHR data and are provided the opportunity to share additional physical measurements and biospecimens at an HPO or DV [21]. The reimbursement procedures are described in the following section.

### Ethical Considerations

The protocol for the AoURP study was reviewed by the institutional review board of the AoURP (protocol 2021-02-TN-001). The institutional review board follows the regulations and guidance of the National Institutes of Health Office for Human Research Protections for all studies, ensuring that the rights and welfare of research participants are overseen and protected uniformly. The informed consent process states that participants have the option to withdraw at any time. Privacy of participant data is maintained in the following three ways: (1) storing data on protected computers, (2) preventing researchers from seeing identifiable patient information, such as name or social security number, and (3) having researchers sign a contract declaring that they will not try to identify participants. Furthermore, access to the AoURP dataset is only available through the Researcher Workbench, which is only accessible to researchers who have completed the requisite training at institutions with a signed data use agreement. For compensation, participants are offered US $25 one-time in the form of cash, gift card, or an electronic voucher if they are asked and decide to go to an *All of Us* partner center for physical measurements to give blood, saliva, or urine samples. Notably, other racial or ethnic groups—including Asian non-Hispanic, Black non-Hispanic, Hispanic or Latinx of any race, more than one population, none of these, and those who skipped the reporting—were not reported in this study because the sample sizes for several of them were less than 20 and could risk patient reidentification, which violates the AoURP Dissemination policy [24].

### Computational Phenotyping of PPD and Non-PPD Cohorts

Females were assigned to the PPD cohort using the same method that we described previously [12,25]. Briefly, identifying females with PPD consists of a three-fold approach: (1) a PPD diagnosis, (2) a diagnosis of depression during the postpartum period, or (3) antidepressant drug exposure during the postpartum period, which is consistent with other computational phenotyping approaches for PPD [26]. Females were assigned to the non-PPD cohort by identifying those with available pregnancy or delivery EHR data in a similar manner to the PPD cohort and then excluding those who were in the PPD cohort.

To assess wear time behavior in a longitudinal manner, Fitbit wear time data for each female in the PPD cohort were assigned to one of the four periods: (1) prepregnancy (starting from 2 years before the PPD index date), (2) pregnancy, (3) postpartum without depression (after the delivery date and before the PPD diagnosis date), or (4) PPD (a diagnosis up to 24 months from the date of delivery, which has been done in previous work and in this study also represents a period) [27,28]. The PPD period ranged from 14 days before the index date to 30 days after the index date, which was selected because (1) the diagnostic criteria for PPD requires displaying 5 depressive symptoms lasting 2 weeks and (2) some individuals received antidepressant medication on the same date as their index date, which can take effect after 4 weeks of use [29,30].

Because females in the non-PPD group did not undergo a *fourth* phase of PPD as seen in the PPD group, we introduced a pseudotime period called the *PPD-equivalent* period as a time frame for the non-PPD group to align with the PPD period. The index date for the PPD-equivalent period was set at 58 days following delivery, corresponding to the median number of days after delivery of PPD diagnosis among females in the PPD group, following the same strategy we used in our previous work. Similarly, 14 days before the index date was not used as these females did not actually experience PPD [12]. Females were only included in the PPD or non-PPD cohorts if they had any Fitbit data during any of the 4 periods.

### Covariates

Models were run with covariates of age at PPD diagnosis (or age at the index date for the non-PPD cohort), race/ethnicity, and annual income, which was determined from self-reported responses in the Basics survey as a measure of socioeconomic status [31]. Potential responses for annual income consisted of the following: (1) <US $10,000, (2) US $10,000-US $24,999, (3) US $25,000-US $34,999, (4) US $35,000-US $49,999, (5) US $50,000-US $74,999, (6) US $75,000-US $99,999, (7) US $100,000-US $149,999, (8) US $150,000-US $199,999, (9) >US $200,000, or (10) prefer not to answer. Notably, previous history of mental health disorders was intentionally excluded as a covariate because its prevalence was significantly higher in the PPD cohort than in the non-PPD cohort. Including it in the model could result in unreliable estimates and therefore we intentionally chose not to adjust for it.

### Outcome of Measuring Fitbit Wear Time in PPD and Non-PPD Cohorts

Fitbit wear time was measured by first determining the number of hours the device was worn in a day using methods described previously [32]. Previous studies have indicated that a *valid* day of smartwatch data requires 10 hours of wear time and between 100 and 45,000 steps [32]. In this study, rather than analyzing days of *valid* data, we wanted to understand patterns of Fitbit wear time behavior among females with PPD. Hence, we established a binary variable for each day to indicate whether the device was worn or not based on the presence of at least 1 hour of wear time, where hours of wear time were measured based on the presence of step data, similar to prior studies [32]. We then determined the percentage of days the Fitbit was worn during each of the 4 periods (ie, prepregnancy, pregnancy, postpartum, and PPD [or PPD-equivalent for the non-PPD cohort]) by counting the number of days the device was worn divided by the total number of days during that period for each female. The total number of days was determined for each person by filtering data after the first recorded date of any Fitbit data to ensure that we were not labeling someone as not wearing their Fitbit if they did not own one at the time.

### Outcome of Measuring the Number of Hours Fitbit Devices Were Worn Per Day in PPD and Non-PPD Cohorts

We determined the number of hours per day Fitbit was worn using the same logic as described in aforementioned section. The dataset was filtered on individuals who had at least 1 hour of wear time, as we wanted to ensure that we were assessing whether there was a difference in the number of hours the device was worn per day among days that the device was actually worn.

### Outcome of Measuring the Percentage of Days Fitbits Were Worn to Sleep in PPD and Non-PPD Cohorts

To assess how often females with PPD wore their device to sleep, we focused on whether females had any record of *main sleep* for each date as a binary variable for yes or no. We then determined the percentage of days the Fitbit was worn to sleep during each of the 4 periods (ie, prepregnancy, pregnancy, postpartum, and PPD [or PPD-equivalent for the non-PPD cohort]) by counting the number of days the device was worn to sleep divided by the total number of days during that period for each female.

### Statistical Analysis Comparing the Percentage of Wear Time, Hours of Wear Time Per Day, and Percentage of Days Fitbits Were Worn to Sleep

The percentage of Fitbit wear time was compared between PPD and non-PPD cohorts using linear regression, where 4 total models were run (one for each period). Models were run with covariates of age at PPD diagnosis (or age at the index date for the non-PPD cohort), race/ethnicity, and annual income. Each model filtered data during one period and the means were calculated using the emmeans() function [33]. Similar methodology was used for comparing the percentage of days Fitbits were worn to sleep among PPD and non-PPD cohorts. For comparing the hours per day of Fitbit wear time between PPD and non-PPD cohorts, we ran a linear mixed-effects model using the lme4 package in R because there were multiple days of data per person (ie, person ID was included as the random effect) [34,35]. All models were run at a significance level of *P*=.05; however, because 4 statistical tests were performed in each analysis (ie, one for each period of pregnancy), we performed Bonferroni correction [36] creating a corrected significance level of *P*=.013.

### Assessing the Correlation Between Device Wear Time Before and During PPD

The correlation between device wear time before and during PPD was performed by filtering on 2 periods of interest (eg, prepregnancy and PPD) and assessing the correlation among all females in the PPD cohort. The *ggpubr* package was used to determine the correlation and was evaluated at a significance level of *P*=.05, which was adjusted to a threshold of *P*=.013 using Bonferroni correction. The same analysis was conducted for comparing device use during pregnancy and PPD periods as well. Both analyses were repeated in females without PPD for comparison.

### Assessing the Correlation Between Device Wear Time Consistency Before and During PPD

Device wear time consistency was measured by determining the maximum number of consecutive days the Fitbit was worn for each period for each unique person. Device wear time for one day was defined using the same definition as before, where we considered an individual wore the device if they had at least 1 hour of wear time for each date based on the presence of step data [32]. We then determined the relationship between device wear time consistency during prepregnancy and PPD by calculating the Pearson correlation coefficient between the maximum number of days the device was worn during the PPD period versus the prepregnancy period at a significance level of *P*=.05, which was adjusted to a threshold of *P*=.013 using Bonferroni correction, using the *ggpubr* package in R [37,38]. We also performed the same analysis replacing the prepregnancy period with the pregnancy period to assess the correlation between device wear time consistency during pregnancy and PPD. This process was repeated in the non-PPD cohort for comparison.

### Sensitivity Analysis Comparing the Percentage of Days Fitbits Were Worn in PPD and Non-PPD Cohorts Across Periods

To validate our results, we conducted a sensitivity analysis using a stricter definition of days worn (≥10 hours, >100 steps, <45,000 steps, consistent with other studies [12,32]) to avoid overestimation of wear time. Similar to methods described in previous sections, for days that met these criteria, we created a binary variable for each day to indicate whether the device was worn or not. We then determined the percentage of days the Fitbit was worn during each of the 4 periods (ie, prepregnancy, pregnancy, postpartum, and PPD [or PPD-equivalent for the non-PPD cohort]) by counting the number of days the device was worn divided by the total number of days during that period for each female. The total number of days was determined for each person by filtering data after the first recorded date of any Fitbit data to ensure that we were not labeling someone as not wearing their Fitbit if they did not own one at the time. A sensitivity analysis was not conducted to compare hours of wear time, as it would not be appropriate to perform a comparison between groups for the number of hours the device was worn only in days it was worn more than 10 hours. We also did not pursue a sensitivity analysis to compare the percentage of days of Fitbit sleep data, as determining whether a device was worn during sleep was based on the presence of *main sleep* Fitbit data rather than step data and hours of wear time.

## Results

### Descriptive Statistics

**Figure 1:**
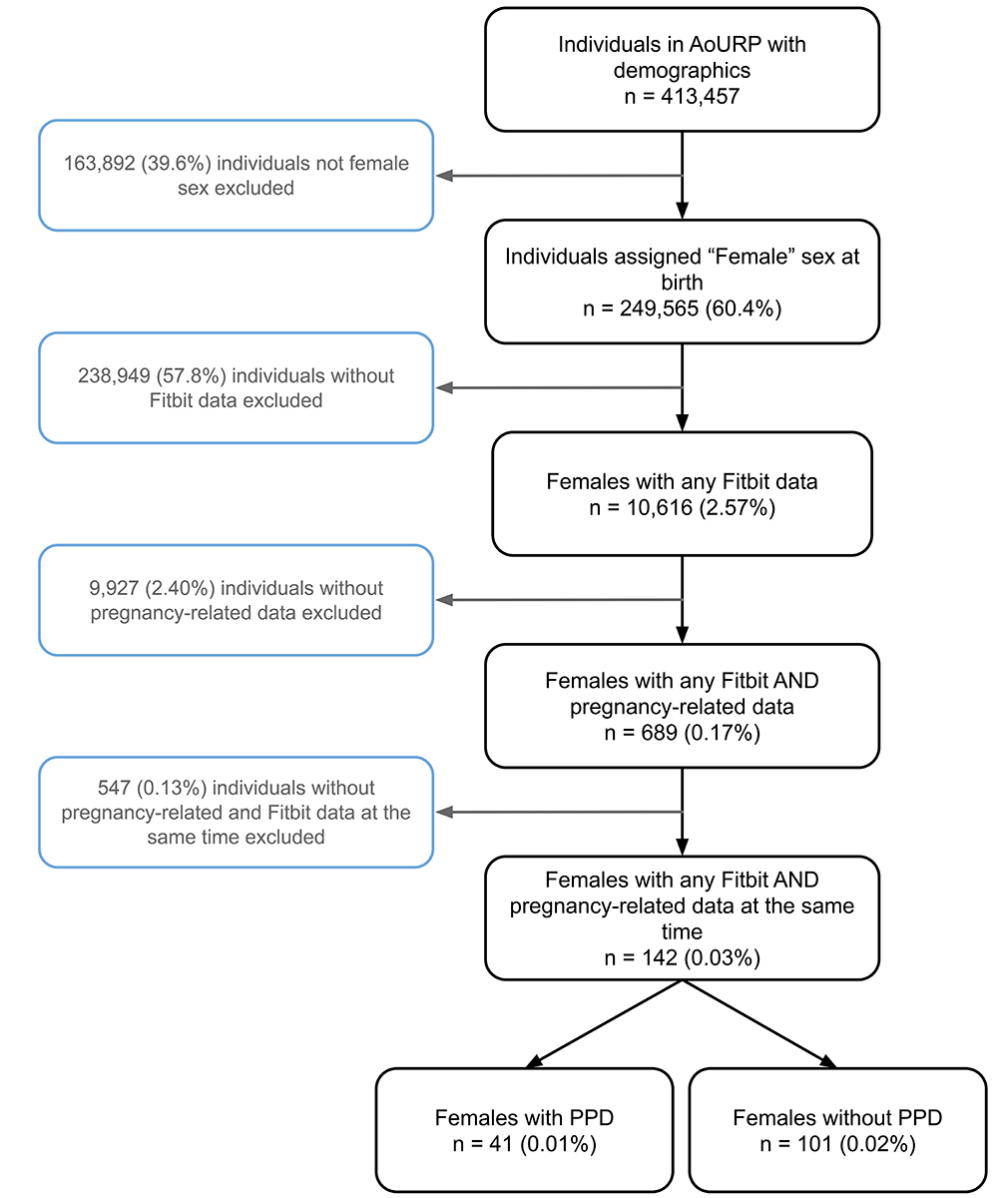
Flow diagram describing the inclusion and exclusion criteria of postpartum depression (PPD) and non-PPD cohorts. AoURP: *All of Us* Research Program.

Females were assigned to the PPD cohort using methods as previously described (Methods section; Figure 1) [12,25]. To summarize, females were assigned to the PPD or non-PPD cohorts using a combination of EHR and Fitbit data. Starting with the entire AoURP longitudinal observational cohort with EHR data, we filtered on (1) individuals assigned female sex at birth, (2) female participants with any Fitbit data (ie, those who already owned a Fitbit and consented to share data), (3) female participants with any pregnancy data, and (4) female participants with Fitbit and pregnancy data at the same time. Females were then assigned to the PPD cohort based on the presence of a PPD diagnostic code, depression diagnostic code during the postpartum period, or antidepressant prescription during the postpartum window using EHR data. The remaining cohort of females not assigned to the PPD cohort were labeled as females without PPD.

Our AoURP cohort consisted of 142 females who experienced pregnancy and had available Fitbit data with a total of 108,062 days of data, where 41 (28.9%) females experienced PPD (31,201 days of data) and 101 (71.1%) females (76,861 days of data) did not. To achieve an accurate comparison in females without PPD, we created a pseudotime period labeled *PPD-equivalent* starting 58 days following delivery, which was the median number of days after delivery for PPD diagnosis (more details about pregnancy periods are provided in the Methods section). In the PPD and non-PPD cohorts, there were 13,225 days and 40,212 days of data during prepregnancy, 11,055 days and 27,559 days of data during pregnancy, 5089 days and 6060 days of data during postpartum, and 1832 days and 3030 days of data during the PPD or PPD-equivalent periods, respectively. The median age of the PPD cohort was 33.1 years (IQR 29.1-35.7 years) compared to 33.9 years (IQR 30.9-37.1 years) for females in the non-PPD cohort. Both the PPD (36/41, 88%) and non-PPD cohorts (76/101, 75.2%) were predominantly White non-Hispanic. Approximately 50% of the PPD cohort and non-PPD cohorts had an annual income of more than US $100,000 (Table 1).

The count and percentage (or median and IQR) of the number of days of Fitbit data during each period of pregnancy; demographics, including age and race/ethnicity; and annual income are provided in Table 1. Periods of pregnancy were defined using EHR data of (1) the PPD index date (14 days before the EHR date of PPD diagnosis to align with the diagnostic requirements of having symptoms for at least 2 weeks), (2) the date of delivery (separating the pregnancy and postpartum periods), and (3) the date of pregnancy (either the first EHR recorded date of pregnancy or 9 months [standard length of pregnancy] before the date of delivery) creating 4 distinct periods of pregnancy. Because females in the non-PPD cohort did not experience PPD, a fourth comparable pseudotime period labeled PPD-equivalent (starting 58 days postdelivery—the median time to PPD diagnosis after delivery in the PPD cohort) was created*.*

**Table 1:** Descriptive statistics of postpartum depression (PPD) and non-PPD cohorts.

Statistics		Cohort		
		Total	PPD	Non-PPD
Number of females, n (%)		142 (100)	41 (28.9)	101 (71.1)
Number of days of data, n (%)		108,062 (100)	31,201 (28.9)	76,861 (71.1)
Number of days of data by period, n (%)				
	Prepregnancy	53,437 (49.5)	13,225 (42.4)	40,212 (52.3)
	Pregnancy	38,614 (35.7)	11,055 (35.4)	27,559 (35.9)
	Postpartum	11,149 (10.3)	5089 (16.3)	6060 (7.9)
	PPD (or PPD-equivalent)	4862 (4.5)	1832 (5.9)	3030 (3.9)
Age (y), median (IQR)		33.7 (30.6-36.5)	33.1 (29.1-35.7)	33.9 (30.9-37.1)
White non-Hispanic females, n (%)		112 (78.9)	36 (87.8)	76 (75.2)
Females with annual income >US $100,000, n (%)		72 (51.1)^a^	<20 (48.8)^a^	52 (51.5)

^a^Note: to comply with *All of Us* Research Program guidelines for counts <20 and mitigate the risk of participant reidentification, the number of females in the PPD cohort and the combined PPD and non-PPD groups with an income >US $100,000 is reported as an estimate.

### Wear Time Patterns in Females With and Without PPD

**Figure 2:**
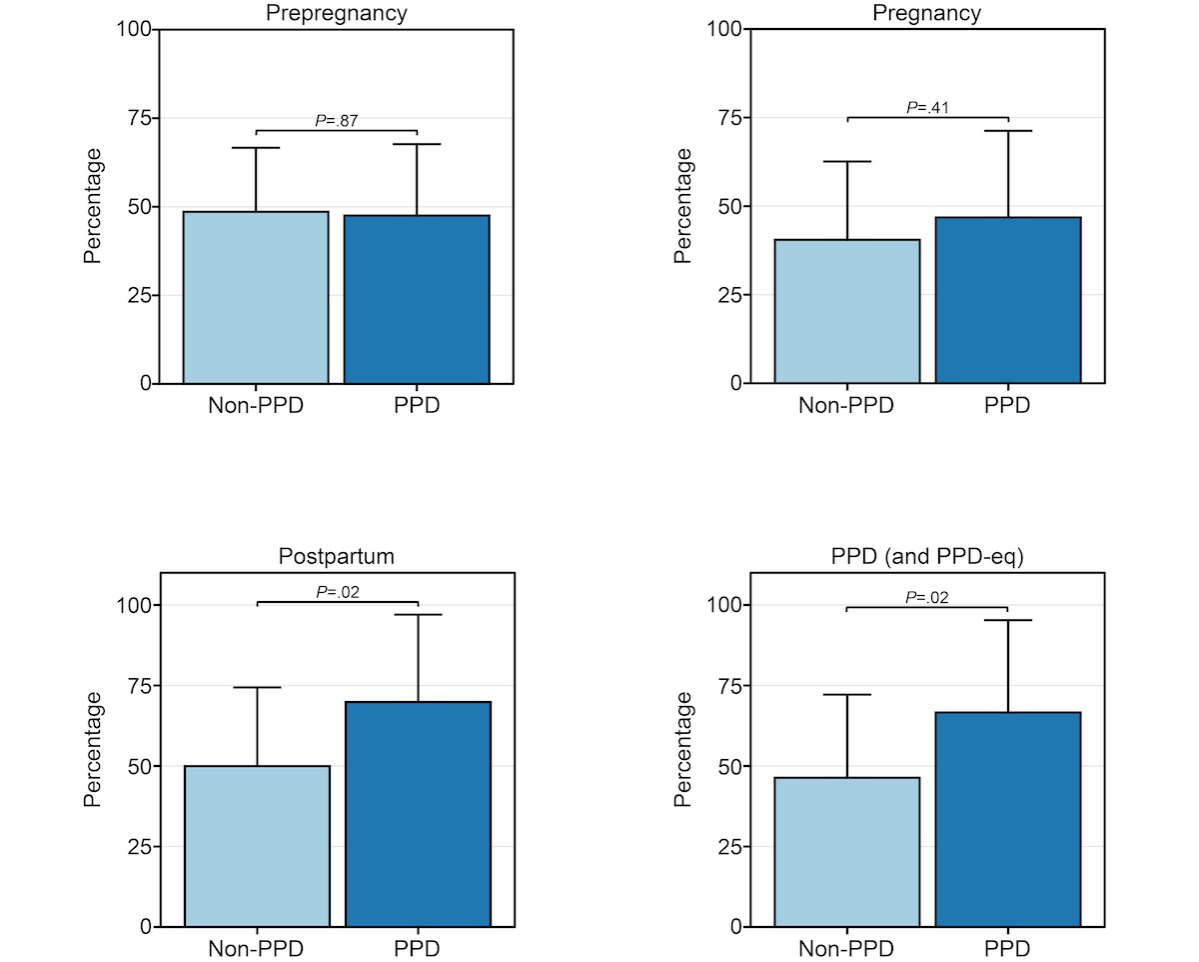
The percentage of days females in the postpartum depression (PPD) and non-PPD *All of Us* Research Program (AoURP) cohorts wore their Fitbit across the prepregnancy (top left), pregnancy (top right), postpartum (bottom left), and PPD (or PPD-equivalent; bottom right) periods. Data in the PPD and non-PPD cohorts were compared using linear regression adjusted for age at PPD diagnosis, race/ethnicity, and annual income and are expressed as mean and 95% CI. PPD-eq: postpartum depression–equivalent.

We first sought to evaluate whether females with PPD displayed Fitbit wear time behavior that differed to those without PPD. We calculated the percentage of days that each female wore their device during the PPD and PPD-equivalent periods and built a linear regression model adjusted for age at PPD diagnosis, race/ethnicity, and annual income. The results revealed a trend (although nonsignificant after using Bonferroni adjusted *P*=.013) that the percentage of days the device was worn in the PPD cohort (mean 66.6%, 95% CI 37.9%-95.3%) was greater than the non-PPD cohort (mean 46.4%, 95% CI 20.5%-72.2%; *P*=.02; Figure 2).

Observing this pattern during the PPD or PPD-equivalent periods, we proceeded to explore potential disparities in wear time between PPD and non-PPD cohorts across other pregnancy stages, including prepregnancy, pregnancy, and postpartum periods. Such analysis aimed to discern potential associations between Fitbit wear time behavior and future PPD onset. Models were run in a similar fashion for the prepregnancy, pregnancy, and postpartum periods, where we also detected a trend, though nonsignificant after multiple testing correction, of increased wear time during the postpartum period, with a mean of 69.9% (95% CI 42.7%-97%) in the PPD cohort compared to 50% (95% CI 25.5%-74.4%) in the non-PPD cohort (*P*=.02; Figure 2). These results suggest that females who go on to develop PPD may wear their device more than those who do not in the postpartum period. Alternatively, there was no significant difference in the percentage of days the device was worn during prepregnancy (PPD cohort: mean 47.5%, 95% CI 27.3%-67.7%; non-PPD cohort: mean 48.6%, 95% CI 30.5%-66.7%; *P*=.87) or pregnancy (PPD cohort: mean 46.8%, 95% CI 22.4%-71.3%; non-PPD cohort: mean 40.5%, 95% CI 18.4%-62.6%; *P*=.41) periods between the 2 cohorts (Figure 2). Sensitivity analysis results showed the same patterns of (1) no difference in Fitbit wear time during prepregnancy (PPD cohort: mean 45.5%, 95% CI 24.1%-66.9%; non-PPD cohort: mean 45.2%, 95% CI 26%-64.4%; *P*=.97) and pregnancy (PPD cohort: mean 44.1%, 95% CI 19.6%-68.5%; non-PPD cohort: mean 38%, 95% CI 15.9%-60.1%; *P*=.44) time periods between PPD and non-PPD cohorts and (2) a trend, though nonsignificant, of increased wear time during the PPD (PPD cohort: mean 64.5%, 95% CI 37%-92%; non-PPD cohort: mean 46.1%, 95% CI 21.3%-70.8%; *P*=.03) and postpartum (PPD cohort: mean 62.6%, 95% CI 36.5%-88.7%; non-PPD cohort: mean 47.8%, 95% CI 24.4%-71.3%; *P*=.06) time periods among females in the PPD cohort compared to females without PPD, with trends slightly less strong than the main analysis (Multimedia Appendix 1).

### Hours Per Day of Wear Time in Females With and Without PPD

**Figure 3:**
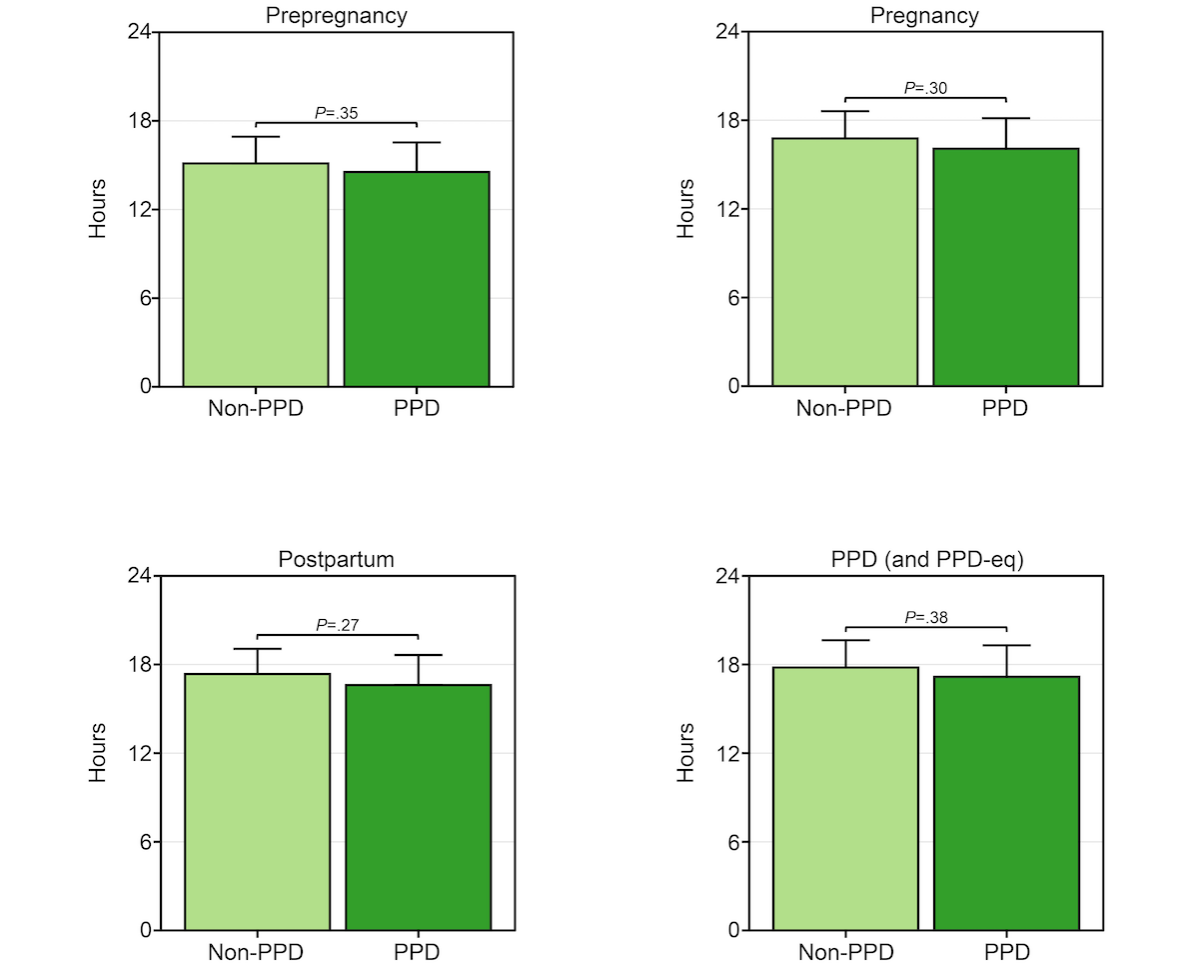
The number of hours per day females in the postpartum depression (PPD) and non-PPD *All of Us* Research Program (AoURP) cohorts wore their Fitbit across the prepregnancy (top left), pregnancy (top right), postpartum (bottom left), and PPD (or PPD-equivalent; bottom right) time periods. Data in the PPD and non-PPD cohorts were compared using linear mixed-effects models with person ID as the random effect adjusted for age at PPD diagnosis, race/ethnicity, and annual income and are expressed as mean and 95% CI. PPD-eq: postpartum depression–equivalent.

Observing variation in the percentage of days wearable devices were worn between PPD and non-PPD cohorts, we subsequently evaluated if there were any differences in the daily duration of device wear time adjusted for age at PPD diagnosis, race/ethnicity, and annual income. Surprisingly, our findings revealed no trends or significant differences between PPD and non-PPD cohorts during prepregnancy (PPD cohort: mean 14.5, 95% CI 12.5-16.5; non-PPD cohort: mean 15.1, 95% CI 13.3-16.9; *P*=.35), pregnancy (PPD cohort: mean 16.1, 95% CI 14-18.1; non-PPD cohort: mean 16.8, 95% CI 14.9-18.6; *P*=.30), postpartum (PPD cohort: mean 16.6, 95% CI 14.6-18.6; non-PPD cohort: mean 17.4, 95% CI 15.6-19.1; *P*=.27), or the PPD or PPD-equivalent periods (PPD cohort: mean 17.2, 95% CI 15-19.3; non-PPD cohort: mean 17.8, 95% CI 16-19.6; *P*=.38; Figure 3).

### Wear Time Patterns to Sleep in Females With and Without PPD

**Figure 4:**
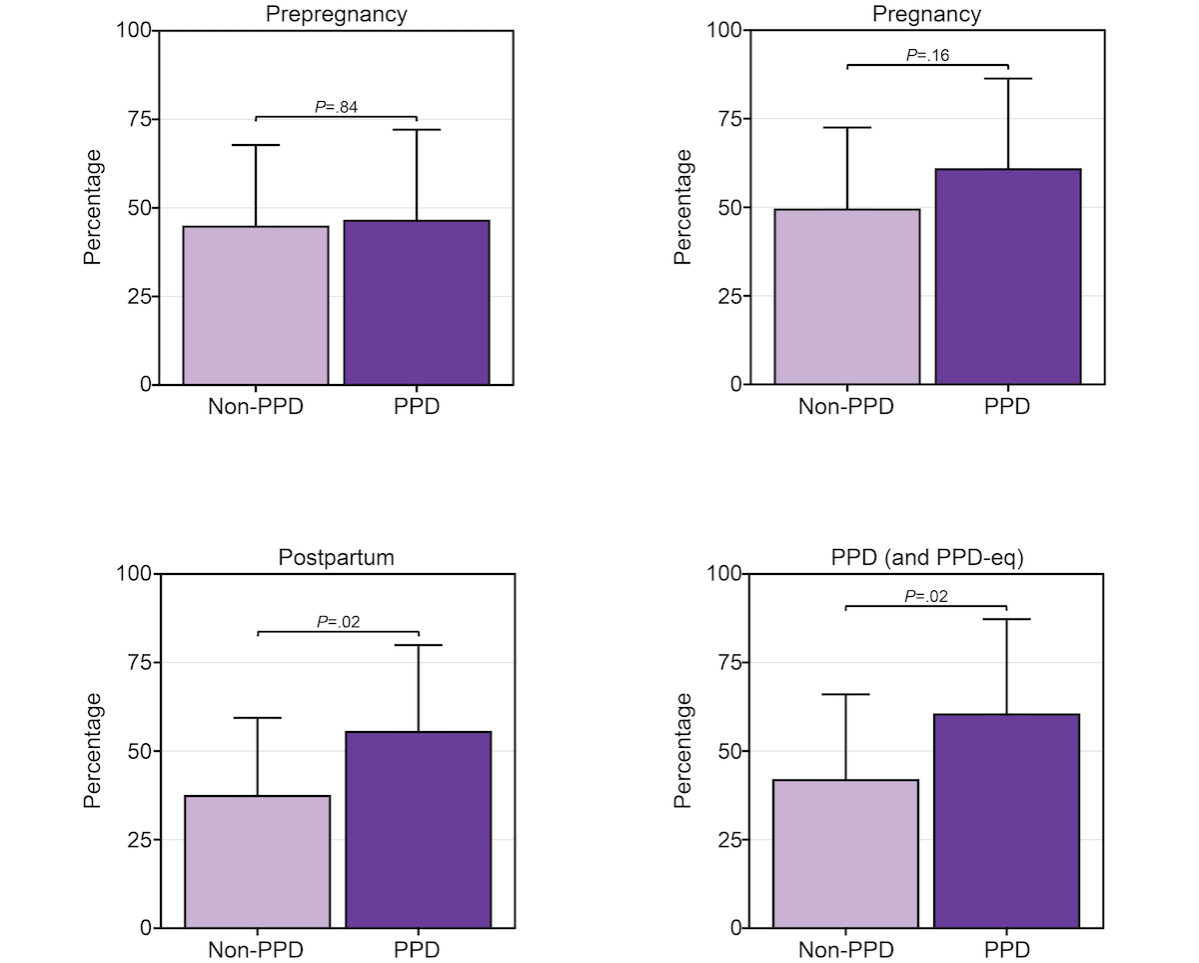
The percentage of days females in the postpartum depression (PPD) and non-PPD *All of Us* Research Program (AoURP) cohorts wore their Fitbit to sleep across the prepregnancy (top left), pregnancy (top right), postpartum (bottom left), and PPD (or PPD-equivalent; bottom right) periods. Data in the PPD and non-PPD cohorts were compared using linear regression adjusted for age at PPD diagnosis, race/ethnicity, and annual income and are expressed as mean and 95% CI. PPD-eq: postpartum depression–equivalent.

Given the extensive connection between sleep and PPD, we sought to describe and compare the percentage of days females in the PPD cohort wore their Fitbit to sleep compared to those without PPD during each phase of pregnancy [39-45]. When comparing the percentage of days females in each cohort wore their Fitbit to sleep, the results showed similar observations to the percentage of wear time findings (Figure 2), where we noticed a trend, though nonsignificant after multiple testing correction, of females wearing the device to sleep more during the postpartum period in the PPD cohort (mean 55.4%, 95% CI 30.8%-79.9%) compared to the non-PPD cohort (mean 37.3%, 95% CI 15.2%-59.4%; *P*=.02) adjusted for age at PPD diagnosis, race/ethnicity, and annual income (Figure 4). There was a similar pattern during the time period females experienced PPD (mean 60.3%, 95% CI 33.4%-87.2%) compared to those who did not (mean 41.8%, 95% CI 17.6%-66%; *P*=.02; Figure 4). No differences were detected in the percentage of days Fitbits were worn to sleep between PPD and non-PPD cohorts during the prepregnancy (PPD cohort: mean 46.4%, 95% CI 20.6%-72.1%; non-PPD cohort: mean 44.7%, 95% CI 21.7%-67.7%; *P*=.84) or pregnancy (PPD cohort: mean 60.7%, 95% CI 35.1%-86.3%; non-PPD cohort: mean 49.4%, 95% CI 26.2%-72.5%; *P*=.16) periods (Figure 4).

### Fitbit Wear Time Consistency During PPD and Other Time Periods of Pregnancy

**Figure 5:**
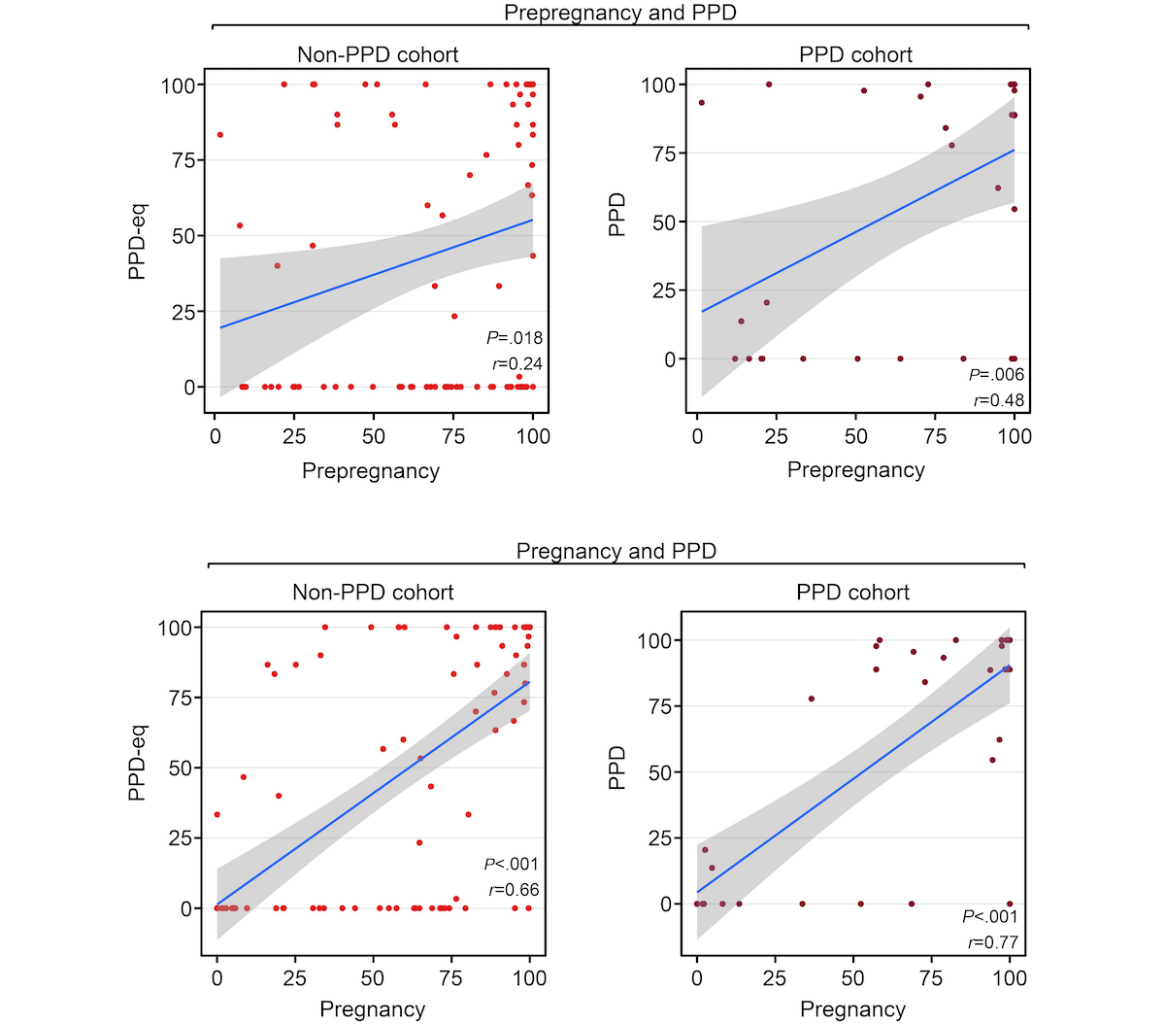
The Pearson correlation coefficient and associated *P* values demonstrate the relationship between the percentage of days females wore their Fitbit across different periods separated by postpartum depression (PPD) status—without PPD (left) or with PPD (right). Top panels display the correlation of females wearing their Fitbit during prepregnancy and PPD periods (or PPD-equivalent for the non-PPD cohort), while bottom panels show the correlation during pregnancy and PPD periods (or PPD-equivalent for those in the non-PPD cohort). The blue line represents the line of best fit and gray shading shows the 95% CI. PPD-eq: postpartum depression–equivalent.

Finally, we wanted to explore individual-level device wear time patterns before and during PPD. Therefore, we examined the correlation between the percentage of days within females who wore their Fitbit during periods before PPD (ie, prepregnancy and pregnancy) with the PPD period. For instance, a positive correlation would suggest that those who wore their Fitbit more frequently during the prepregnancy period tended to do so during the PPD period. We conducted this analysis in parallel with the non-PPD cohort for comparison. In females with PPD, the results displayed a significant positive correlation between the percentage of days the Fitbit was worn during prepregnancy and PPD periods (*r*=0.48, 95% CI 0.16-0.71; *P*=.005; Figure 5). A positive correlation was also detected during prepregnancy and PPD-equivalent periods among females without PPD, but it did not reach statistical significance after Bonferroni correction (*r*=0.24, 95% CI 0.04-0.42; *P*=.02; Figure 5). There also existed a strong positive correlation between the percentage of wear time during the pregnancy and PPD periods among females in the PPD (*r*=0.77, 95% CI 0.59-0.88; *P*<.001) and non-PPD cohorts (*r*=0.66, 95% CI 0.53-0.76; *P*<.001; Figure 5).

**Figure 6:**
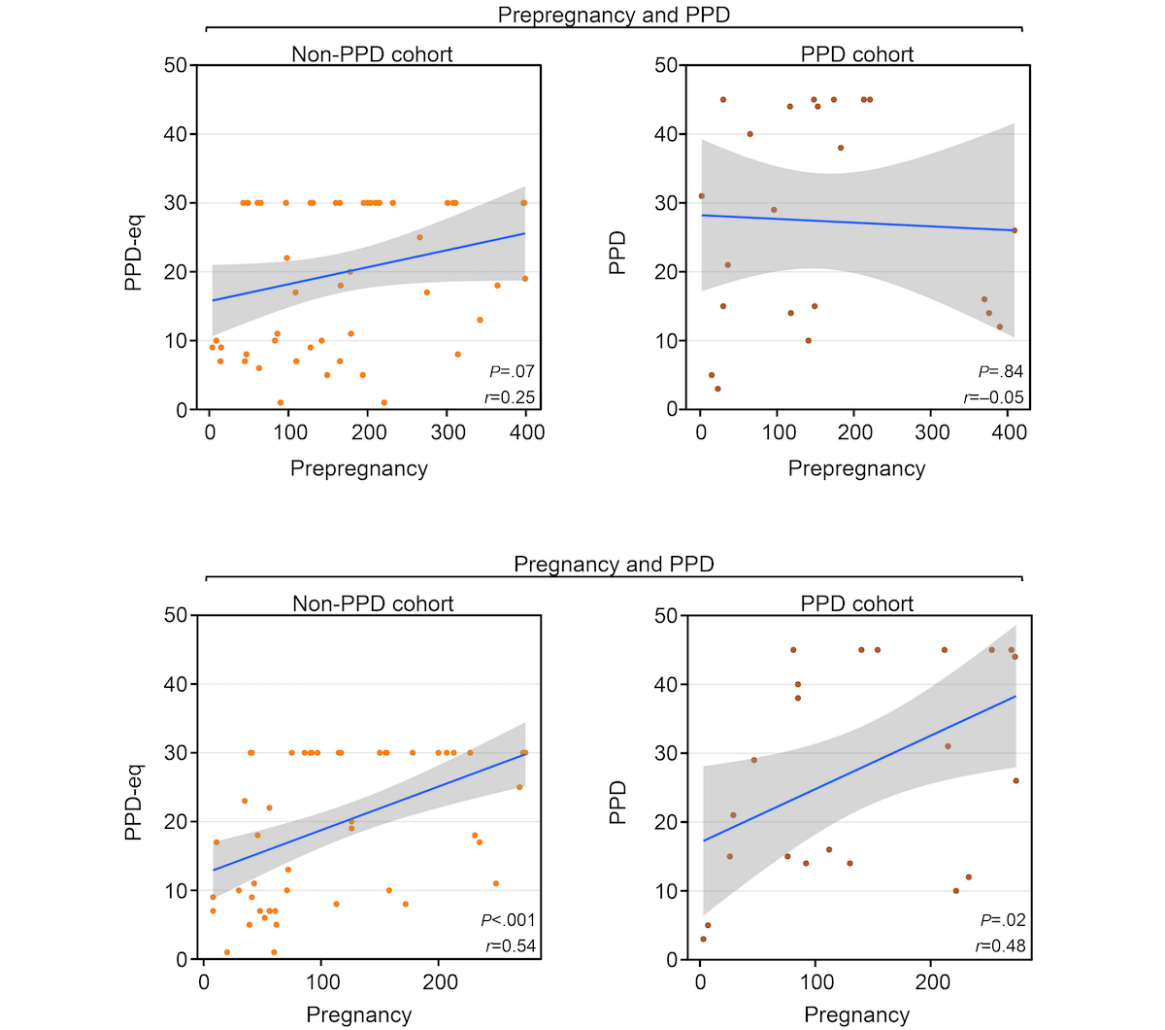
The Pearson correlation coefficient and associated *P* values demonstrate the relationship between the maximum number of days in a row (ie, consistency) females wore their Fitbit across different periods separated by postpartum depression (PPD) status—without PPD (left) or with PPD (right). Top panels display the correlation of females wearing their Fitbit during prepregnancy and PPD periods (or PPD-equivalent for the non-PPD cohort), while bottom panels show the correlation during pregnancy and PPD periods (or PPD-equivalent for those in the non-PPD cohort). The blue line represents the line of best fit and gray shading shows the 95% CI. PPD-eq: postpartum depression–equivalent.

To further understand the Fitbit wear time behavior of females during each period, we also sought to analyze the consistency with which Fitbit devices were worn. Our earlier analyses focused on comparing the percentage of days the device was worn across different periods; however, we acknowledged that wear patterns could vary. For example, if there were 50 days in total to potentially wear the device during one of the periods, wearing it consistently for 25 consecutive days followed by 25 days of nonuse is different from alternating between wearing and not wearing the device every other day, even though both scenarios indicate 50% wear time. Therefore, to assess individual-level consistency during each period, we determined the maximum consecutive number of days the device was worn during each period and examined the correlation across periods (ie, during prepregnancy and PPD [or PPD-equivalent] and during pregnancy and PPD [or PPD-equivalent]). The results displayed a trend, though nonsignificant, in wear time consistency between prepregnancy and PPD-equivalent periods among females without PPD (*r*=0.25; *P*=.07), while those with PPD did not exhibit any correlation (*r*=–0.05, 95% CI –0.46 to 0.38; *P*=.84; Figure 6). Alternatively, a significant correlation was present in the non-PPD (*r*=0.54, 95% CI 0.31-0.7; *P*<.001) cohort between the pregnancy and PPD (or PPD-equivalent) periods, but only a strong trend, which was nonsignificant after multiple testing correction, was observed in the PPD cohort between the 2 periods (*r*=0.48, 95% CI 0.08-0.74; *P*=.02; Figure 6). These data suggest a relationship between the consistency of Fitbit use during pregnancy and the PPD (or PPD-equivalent) periods in both cohorts. Notably, when analyzing the consistency of Fitbit wear time during prepregnancy, the relationship only was present among females in the non-PPD cohort (Figure 6).

## Discussion

### Principal Findings

Our study elucidated numerous insights related to Fitbit wear time and PPD across periods of pregnancy. First, we observed a trend, though nonsignificant after multiple testing correction, that females with PPD wear their device a higher percentage of days than those without PPD during the postpartum and PPD periods (Figure 2). However, among the days that females with and without PPD wore their device, there was not a significantly different number of daily hours the device was worn (Figure 3). Regarding Fitbit wear time during sleep, a similar pattern was observed as the percentage of daily wear time. Females in the PPD cohort wore their Fitbit to sleep on a higher percentage of days during the postpartum and PPD periods compared to those in the non-PPD cohort (Figure 4). It was also observed that females in the PPD and non-PPD cohorts displayed the same correlation patterns between wear time during earlier and later pregnancy periods (Figure 5). Finally, we found that females in both cohorts who wore their devices more consistently during pregnancy also maintained higher levels of device wear consistency during the PPD (or PPD-equivalent) periods. However, there was no correlation in the consistency of Fitbit wear time during the prepregnancy and PPD periods among females in the PPD cohort (Figure 6).

Our study’s first key finding showed that females with PPD wore their Fitbit a higher percentage of days compared to females without PPD. A similar pattern was detected during the postpartum periods among females from PPD and non-PPD cohorts (Figure 2). The trend was not as strong in our sensitivity analysis using a stricter definition of Fitbit wear time (Multimedia Appendix 1). One reason we hypothesize that females with PPD wore their devices more frequently than those without PPD is due to anxiety and hypervigilance, which commonly occurs in females with PPD, and may drive increased personal tracking behavior [46]. Because PPD often goes undetected, females in the PPD cohort during the postpartum period (before EHR diagnosis) may have already been experiencing PPD symptoms, which could explain the similarities in patterns observed between the postpartum and PPD periods [4]. Unfortunately, AoURP does not have symptom-related data so we cannot know for sure when symptoms began, which could also explain the differences in our analyses during the postpartum period where some females may have been experiencing symptoms while others were not. While it was originally suggested that PPD symptoms peak between 4 and 6 weeks in the postpartum period [47,48], recent work suggests subgroups of females display unique symptom trajectories [49]. Furthermore, although females in the PPD cohort tended to wear their Fitbit a higher percentage of days during the postpartum and PPD periods, we did not detect any difference in the number of hours per day the device was worn compared to females without PPD (Figure 3). Our findings showed that both the PPD and non-PPD cohorts wore their devices approximately 15 to 17 hours of the day, which is consistent with other studies involving Fitbits [50,51]. Our findings also align with previous research in perinatal populations, where (1) Sarhaddi et al [52] reported that women wore their wearable devices for an average of 17 hours per day during pregnancy and 13.7 hours per day postpartum (up to 12 weeks after delivery) and (2) Grym et al [53] reported device wear time for an average of 17.3 hours per day during pregnancy and 14.4 hours per day postpartum (up to 4 weeks after delivery). These findings are also similar to individuals with depression who wore their device for an average of 15 to 17 hours per day [54].

The next component of our study was to investigate the percentage of days females with PPD wear their device to sleep across each phase of pregnancy compared to those without PPD given the extensive relationship between sleep and PPD [39-45]. Our findings revealed that females in the PPD cohort tended to wear their device more to sleep during the postpartum and PPD periods compared to those without PPD (Figure 4). Considering the similar pattern observed in Fitbit wear time frequency (Figure 2), it was not surprising to find the same result in sleep data. Unfortunately, it is not possible to determine whether the device was intentionally worn for sleep tracking or simply due to continued use, but it could be interesting to investigate this in future studies. The fact that females on average wear their device between 15 and 17 hours per day suggests that when females in these cohorts do wear their device, they also wear it to sleep [50,51].

Finally, our study sought to assess whether Fitbit wear time behavior during periods before PPD may correlate with behavior during PPD, with the potential that wear time behavior during prepregnancy or pregnancy periods may be able to help predict PPD onset. Our findings displayed that females who wore their device more during prepregnancy also wore their device more during PPD (Figure 5). A similar observation was detected when comparing pregnancy and PPD periods; however, this relationship also persisted among females in the non-PPD cohort (Figure 5). When assessing the consistency of Fitbit wear time, we noticed a trend, though nonsignificant, only in females without PPD that greater wear time consistency in prepregnancy correlated with greater consistency during the PPD-equivalent period (Figure 6). This may be attributed to females with PPD experiencing cooccurring mood and anxiety symptoms, leading them to wear their devices more frequently regardless of prepregnancy consistency [46]. In addition, wear time patterns of wearable device during prepregnancy and PPD periods findings may also be due to behavioral or lifestyle factors, such as increased motivation for sleep tracking, as wearables increase perception of sleep quality and are increasingly used for sleep assessment, which is important for new mothers [55-57].

### Limitations

While this study is the first to evaluate wear time behavior of wearable device among females with PPD across phases of pregnancy, it is not without limitations. First, the number of hours the device was worn was estimated based on recorded Fitbit steps data using previously established methods; however, it is not *ground truth* data and therefore may contain some level of inaccuracy [32]. We estimated the hours per day (and percentage of days) females in the PPD and non-PPD cohort wore their Fitbit based on the presence of step data, which is consistent with our previous work and that of others [12,32]. Unfortunately, it is not possible to know exactly how long someone wore their device from the AoURP retrospective data. Second, we do not have access to study participants in AoURP to perform any qualitative analysis to further understand causal relationships about individual-level Fitbit wear time patterns and disease symptoms or severity. Wear time could be impacted by factors not controlled for in the study, such as skin issues from using a wristband, breastfeeding status (which was not available in AoURP), and mental health history (which was intentionally excluded due to its significantly higher prevalence in the PPD cohort compared to the non-PPD cohort, and including it in the model could result in unreliable estimates) [47,58,59]. Future studies should include user-experience-related questionnaires and qualitative methods tailored toward women with and without PPD during the postpartum period to better assess the causality between PPD, hypervigilance, other potential confounding factors, and device wear time [15-19]. Third, this study only investigated wearable device behavior for Fitbit. While Fitbit is the most commonly wearable device used for research purposes, it would be valuable to incorporate other devices, such as the Apple Watch, Google Fit, Garmin smartwatch, or Oura ring, which have shown high levels of adherence, and the type of device could be adjusted as a covariate [60-63]. Fourth, the PPD and non-PPD cohorts were relatively small, and we posit we may have observed statistical significance with larger sample sizes. Furthermore, future studies with larger sample sizes should include a sensitivity analysis in females with a confirmed PPD diagnosis to robustly validate our findings to address potential overclassification of PPD diagnosis, which we could not do in this study as AoURP Data and Statistics Dissemination policy prohibits analyses on samples sizes less than 20 individuals to protect patient privacy [24]. Finally, there was likely a selection bias, because our cohorts consisted primarily of females who were White and non-Hispanic. The lack of racial or ethnic diversity may influence study results and limit their generalizability to other patient populations. Previous studies have shown racial or ethnic disparities in PPD prevalence, such as the substantial increase in PPD among Asian and Pacific Islanders and Black non-Hispanic women [64]. Future work should replicate our study findings in diverse populations to evaluate differences in wear time behavior of a wearable device between PPD and non-PPD cohorts across different racial or ethnic groups with additional confounding variables (eg, mental health history and breastfeeding status), if available. We want to clarify that all Fitbit data used in this study was collected by AoURP, where the program operates under a bring-your-own-device model and participants who consent to share Fitbit data already own their device [22]. Notably, one strength of this study is that AoURP does not send any type of notification or reminders for continued use, thus our work provides a great foundation for the first study to assess real-world wear time behavior of a wearable device in females with PPD.

### Interpretation

PPD remains underscreened and consequently underdiagnosed for several reasons, such as its heterogeneity and stigmatization [8]. Wearable devices offer a promising avenue for continuous mental health monitoring and early detection through their ability to capture high-density longitudinal data, including physical activity and sleep patterns—factors known to influence PPD [45,55]. Our findings showed that females with PPD exhibit higher wear time of a wearable device compared to those without PPD, suggesting that wear time patterns may serve as an additional digital biomarker beyond traditional sensor data. While wear time alone may not be specific enough to detect PPD, its potential clinical value lies in its integration with other wearable digital biomarkers, such as longitudinal patterns of physical activity and sleep. Building on our previous work that demonstrated individualized ML models using wearable sensor data (eg, day-level average heart rate, sum of steps, and activity calories) can differentiate PPD from other pregnancy periods, the results of this study suggest that combining sensor data with wear time patterns could enhance PPD detection capabilities [12]. We propose that future clinical implementation of PPD detection ML algorithms using wearables can include wear time of a wearable device in addition to sensor-derived digital biomarkers to prompt PPD screening using the EPDS [9]. However, challenges such as ensuring data privacy, usability, providing adequate clinician training, acceptability to clinicians, improving patient accessibility, and compatibility with existing screening tools (eg, EPDS) must be addressed, along with fostering collaboration between maternal and mental health services to create dedicated care pathways for comprehensive perinatal support [9,59,60,65]. To eventually achieve the goal of clinical implementation, additional work is needed using advanced ML approaches to quantify the relative importance of wear time of a wearable device compared to other features in PPD prediction models, ultimately working toward more effective early screening and diagnosis protocols.

### Conclusions

Understanding wear time behavior of a wearable device can provide insightful clinical information related to women with PPD. Considering that screening and diagnosis of PPD pose significant challenges, wearables, including features of wear time behavior, could potentially offer a viable solution. We envision a future using wearables combined with an ML algorithm that incorporates wear time of a wearable device with other digital biomarkers, such as sleep and physical activity, to facilitate early detection of PPD by notifying the clinician with potential concern to prompt timely screening. Wear time behavior presents a passive and relatively straightforward feature to aid in evaluating PPD in nonclinical environments, and its application could potentially extend to other perinatal and general mental health disorders.

## Data Availability

The datasets generated or analyzed during this study are not publicly available due to data security and privacy guidelines defined by *All of Us* Research Program (AoURP). However, access to the entire AoURP cohort is available to those who (1) complete the required training activities requested by AoURP and (2) create an account on the Researcher Workbench. To individuals who complete the necessary requirements in AoURP, data and code for this study are available upon reasonable request to the corresponding author.

## Appendix

Multimedia Appendix 1 The percentage of days females in the PPD and non-PPD AoURP cohorts wore their Fitbit using a stricter definition of wear time. AoURP: *All of Us* Research Program; PPD: postpartum depression.

## Abbreviations

AoURP: *All of Us* Research Program
DV: direct volunteer
EHR: electronic health record
EPDS: Edinburgh-Postnatal Depression Scale
HPO: health care provider organization
ML: machine learning
PPD: postpartum depression
