# Supplementary material for: Unlocking the Potential of Wear Time of a Wearable Device to Enhance Postpartum Depression Screening and Detection: Cross-Sectional Study
Source: JMIR Form Res. 2025 May 23;9:e67585. doi: 10.2196/67585 (PMC12144471; doi:10.2196/67585)
Supplement: Multimedia Appendix 1 [file formative_v9i1e67585_app1.docx]

**Supplementary Results**

**Figure S1: Females in the PPD cohort tended to wear their wearable device more than those in the non-PPD cohort during the postpartum and PPD time periods using a stricter definition of wear time.**

**
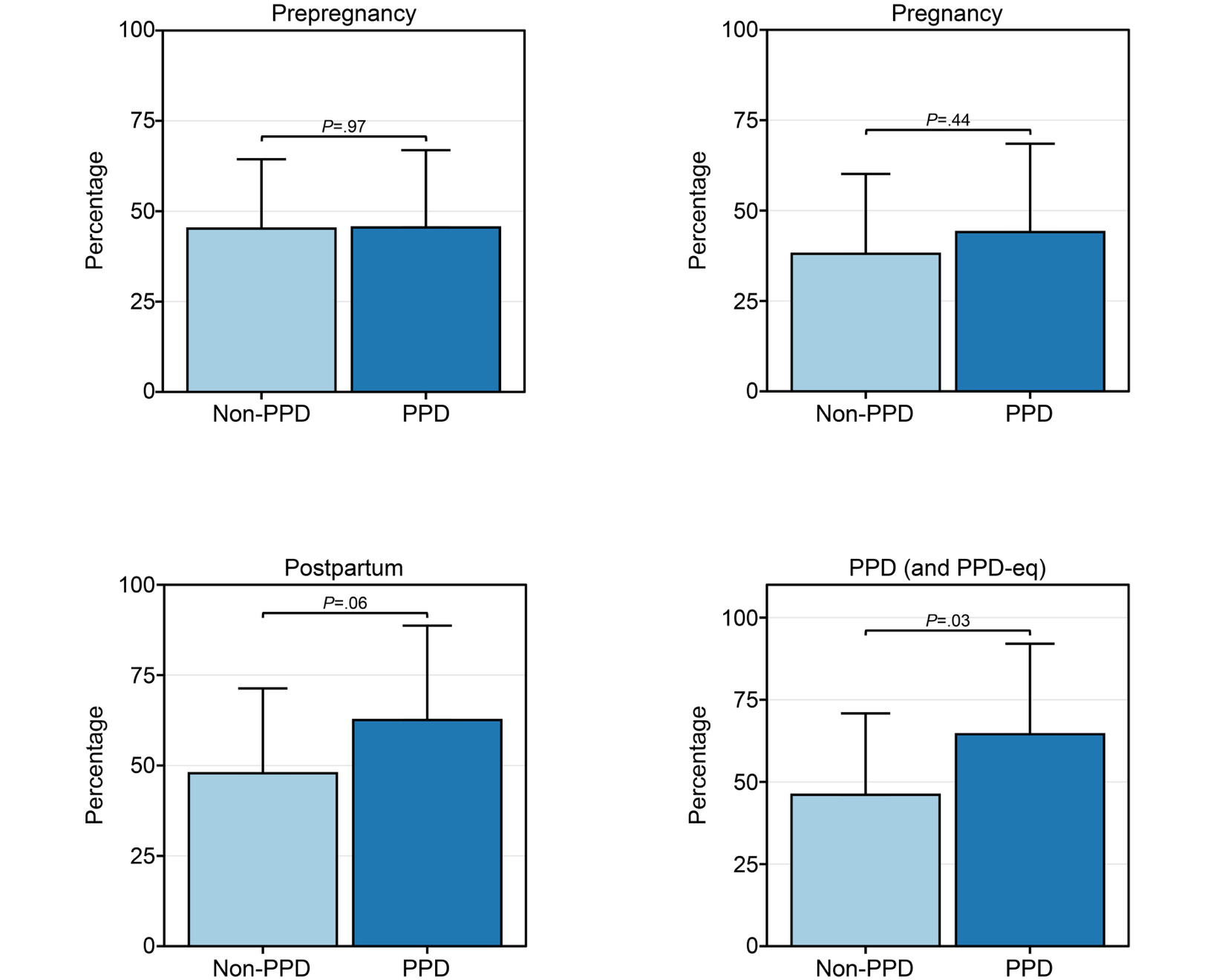
**

PPD-eq = PPD-equivalent

*The percentage of days females in the PPD and non-PPD AoURP cohorts wore their Fitbit (days worn was defined using a stricter definition of ≥10 hours, >100 steps, <45,000 steps) across the prepregnancy (top left), pregnancy (top right), postpartum (bottom left), and PPD (or PPD-equivalent; bottom right) time periods. Data in the PPD and non-PPD cohorts were compared using linear regression adjusted for age at PPD diagnosis and race/ethnicity and are expressed as mean and 95% confidence interval.*
